# Identification of Potential High-Risk Habitats within the Transmission Reach of *Oncomelania hupensis* after Floods Based on SAR Techniques in a Plane Region in China

**DOI:** 10.3390/ijerph14090986

**Published:** 2017-08-30

**Authors:** Yuanyuan Shi, Juan Qiu, Rendong Li, Qiang Shen, Duan Huang

**Affiliations:** 1 Key Laboratory of Monitoring and Estimate for Environment and Disaster of Hubei Province, Institute of Geodesy and Geophysics, Chinese Academy of Sciences, Wuhan 430077, China; shiyuanyuan13@mail.ucas.ac.cn (Y.S.); qiujuan2011@gmail.com (J.Q.); cl980606@whigg.ac.cn (Q.S.); huangduan@asch.whigg.ac.cn (D.H.); 2 University of Chinese Academy of Sciences, Beijing 100049, China

**Keywords:** schistosomiasis, *Oncomelania hupensis*, flooding, SAR, dispersal range, snail habitats

## Abstract

Schistosomiasis japonica is an infectious disease caused by *Schistosoma japonicum*, and it remains endemic in China. Flooding is the main hazard factor, as it causes the spread of *Oncomelania hupensis*, the only intermediate host of *Schistosoma japonicum*, thereby triggering schistosomiasis outbreaks. Based on multi-source real-time remote sensing data, we used remote sensing (RS) technology, especially synthetic aperture radar (SAR), and geographic information system (GIS) techniques to carry out warning research on potential snail habitats within the snail dispersal range following flooding. Our research result demonstrated: (1) SAR data from Sentinel-1A before and during a flood were used to identify submerged areas rapidly and effectively; (2) the likelihood of snail survival was positively correlated with the clay proportion, core area standard deviation, and ditch length but negatively correlated with the wetness index, NDVI (normalized difference vegetation index), elevation, woodland area, and construction land area; (3) the snail habitats were most abundant near rivers and ditches in paddy fields; (4) the rivers and paddy irrigation ditches in the submerged areas must be the focused of mitigation efforts following future floods.

## 1. Introduction

Schistosomiasis caused by *Schistosoma japonicum* is a type of parasitic disease. It is endemic in 12 provinces south of the Yangtze River in China, and millions of people are infected [1,2]. The distribution of *Oncomelania hupensis*, the only intermediate host of *Schistosoma japonicum*, corresponds closely to the epidemic area of *Schistosoma japonicum* [3,4]. The geographic distribution of *Oncomelania hupensis* is closely related to climate and environmental factors, including temperature, rainfall, latitude, elevation, hydrology, soil and vegetation, which affect its growth and reproduction [1,5]. Among these important factors, abundant rainfall and frequent floods facilitate the diffusion of *Oncomelania hupensis*, which increases the prevalence of *Schistosoma japonicum* infection in humans [2,6,7,8,9,10,11]. Therefore, identifying the areas at risk of possible dispersal of *Oncomelania hupensis* after torrential rains and simulating the potential high-risk habitats are of great importance in forecasting the transmission of schistosomiasis.

Numerous studies have studied the impact of floods on the dispersal of *Oncomelania hupensis* and the potential habitats of *Oncomelania hupensis* separately [11,12,13]. Studies have rarely combined these aspects to determine the potential high-risk habitats within the transmission reach of *Oncomelania hupensis* after floods. In previous studies, the application of remote sensing Landsat TM data has been used to measure the impact of flooding on the dispersal of *Oncomelania hupensis* or to identify its habitats [14,15,16,17]. Compared with Landsat TM satellite data, synthetic aperture radar (SAR) is a more powerful source of data with a wide swath, short re-visit cycle, all-weather capability and high resolution. This technique uses a microwave band to obtain accurate images under all weather conditions, including cloudy and rainy days and at night, whereas optical sensors cannot penetrate clouds. SAR time series data have been successfully used to simulate flood inundation, map flood dynamics, and predict water levels [18,19,20]. Sentinel-1A, the first satellite in the European Space Agency’s Copernicus program for monitoring the environment, was launched in April 2014 and carries a C-band radar system. Its radar images are now available routinely every 12 days and are systematically used for land monitoring. Sentinel-1A data have been used in studies to determine waterlines, to estimate ocean wave heights and to assess grassland yields [21,22,23]. SAR data from Sentinel-1A before and during a flood can be used to extract the flood-inundated areas fast and effectively; however, these data have rarely been used in previous *Oncomelania hupensis* research. Flood-inundated areas are usually accompanied by snail dispersal due to the hydrologic regime changes. The potential range of the snail dispersal after a flood can be determined by predicting the potential snail habitats of the submerged zone.

Remote sensing technologies, geographic information system (GIS) spatial analysis tools and statistical analysis methods provide technical support for the analysis of geography-related factors and the simulation and prediction of the spatial distribution of *Oncomelania hupensis* [24,25]. Landscape patch analysis metrics of patches, edges and corridors are a good way to investigate the environmental determinants of disease transmission [13,26,27], and this type of analysis has been applied successfully to study the effects of a number of environmental variables on intermediate hosts [28,29,30]. Statistical analysis methods have achieved good results in spatial distribution estimates and simulations in environmental research [31,32]. These analytical methods and techniques can be used to explore natural environmental factors and to analyze and predict the areas at risk of *Oncomelania hupensis* after floods.

Thus, the main objectives of this study were to: (1) investigate the applicability of Sentinel-1A radar data for snail research, (2) identify areas at risk of dispersal of *Oncomelania hupensis* after torrential rains and (3) forecast potential high-risk snail habitats within the transmission reach. Results of this study will provide scientific guidance for *Oncomelania hupensis* control after flooding.

## 2. Materials and Methods

### 2.1. Study Area

**Figure 1:**
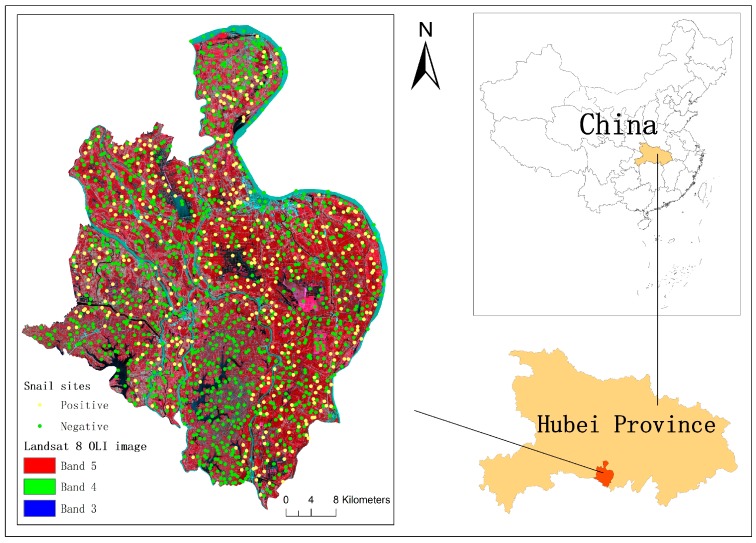
The location of Gongan County and the distribution of snail sample sites overlaid on a satellite image.

Gongan County (Figure 1) in Hubei Province is located on the southern bank of the Yangtze River and has been one of the most severe endemic areas for schistosomiasis in China. It has a humid subtropical monsoon climate and four distinct seasons with a short frost period, long sunshine period and abundant rainfall. Gongan County is primarily composed of plains and lake regions, with an average elevation of approximately 36 m above sea level. These climatic and geographical attributes of Gongan County have created hospitable habitats for *Oncomelania hupensis* to grow and breed. In 2015, *Oncomelania hupensis* was detected in 205 villages among a total of 354 administrative villages of Gongan County.

### 2.2. Data Sources

All relevant input data used in this study are shown in Table 1. Sentinel-1A data collected on 9 June 2016 and 9 July 2016, before and after a flood, respectively, were obtained from the Sentinel-1 Scientific Data Hub (https://scihub.esa.int). Both images were high-resolution (10 m) V-H polarized C-band data acquired in the interferometric wide swath mode. A multispectral image of Landsat 8 OLI over Gongan County, taken on 30 June 2016, was obtained from the Geospatial Data Cloud. The data on *Oncomelania hupensis* were based on annual field investigation at the administrative village scale performed by health professionals in 2015, which were official *Oncomelania hupensis* data from Hubei Provincial Center for Disease Control and Prevention. The land use data of Gongan County, with accurate classifications from 2013 to 2015, and the soil texture data in the study area, i.e., the proportions of silt, sand and clay, were acquired from the Resources and Environmental Science Data Center of the Chinese Academy of Sciences. The elevation data were from the Advanced Spaceborne Thermal Emission and Reflection Radiometer Global Digital Elevation Model (ASTER GDEM) product with a 30-m resolution, as provided by the Computer Network Information Center, Chinese Academy of Science.

### 2.3. Data Processing

**Figure 2:**
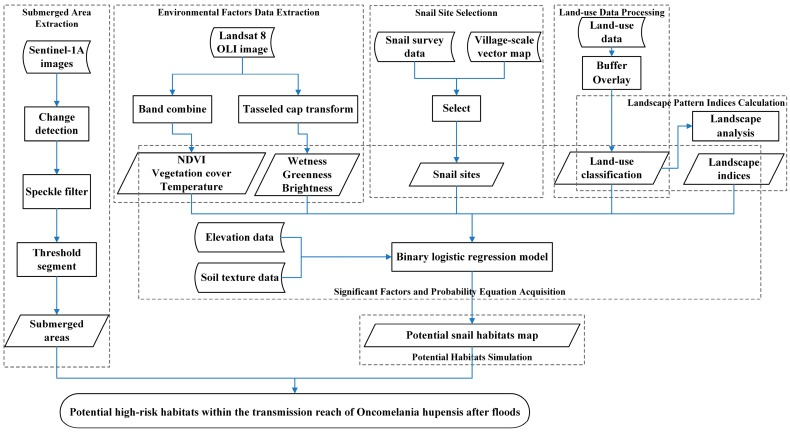
Data processing steps and technology roadmap.

The data processing to determine the potential high-risk habitats within the transmission reach of *Oncomelania hupensis* after floods consisted of seven parts: (1) submerged area extraction; (2) environmental factor data extraction; (3) snail site selection; (4) land use data processing; (5) landscape pattern index calculation; (6) significant factor and probability equation acquisition; and (7) high-risk potential habitat simulation. The process is illustrated in Figure 2.

#### 2.3.1. Submerged Area Extraction

As the process of submerged area extraction shown in Figure 2, the two Sentinel-1A images were processed by change detection first [33]. Then we used speckle filter to filter the results by lee sigma filtering method. After that, we segmented the change threshold. From the log ratio image statistics, it can be found that 95% of the observed value (log ratio) in the range from −0.84 to 0.84. Due to precipitation, the radar had a low scattering coefficient. Lower pixel values were reflected on the image. Therefore, we selected pixels with pixel value above 0.84 as significant changes in the region. That is, the ratio of the two images before and after the flood is greater than 1.8 pixels as a significant area of change. After geocoding the extracted results, we finally obtained the submerged areas.

#### 2.3.2. Environmental Factor Data Extraction

Most Vegetation indices, including the leaf area index (LAI), fractional vegetation cover, and biomass [34,35,36,37], derived from satellite images are based on algebraic combinations of reflectance in the red, R, and near-infrared, NIR, spectral bands [38,39,40,41]. After atmospheric correction through Spectral Hypercubes (FLAASH) module, the normalized difference vegetation index (NDVI), fractional vegetation cover (Fv) and land surface temperature (LST) were extracted from Landsat 8 image using ENVI 5.3 software (Exelis Inc., Boulder, CO, USA) via algebraic band combinations [42,43,44,45].

And the wetness index, greenness index, and brightness index were calculated via tasseled cap transformation. The tasseled cap transformation, constructed by Kauth and Tomas in 1976 [46], is a useful tool for incorporating more information into vegetation indices by using the original six different bands of the TM image associated with physical scene characteristics [47]. In general, the resulting first three features, i.e., brightness, greenness, and wetness, contain the vast majority of the information [47]. The first feature, brightness, measures soil brightness, or total reflectance. The second feature, greenness, reflects vegetation spectral information. The third feature, wetness, primarily expresses the soil moisture status. The resolution of the basic spatial unit of environmental factor data extraction is 30 m.

#### 2.3.3. Snail Site Selection

Based on the spatial database of snail established by combining the *Oncomelania hupensis* village-scale survey data sheets, village-scale vector map and detailed distribution map, samples were chose. In total, 1602 snail sites were randomly selected in Gongan County: 443 snail-positive (present) sites were selected in the snail area and 1159 snail-negative (absent) sites were randomly selected throughout non-snail area based on two conditions [48]: (i) being at a minimum distance of 100 m from any positive site to prevent falling in the snail area; and (ii) being at a minimum distance of 400 m between two negative sites to avoid aggregation. The different numbers for positive and negative sites is a random result for the samples random and well-distributed. Accordingly, 75% of snail sites (i.e., 334 snail-positive sites and 886 snail-negative sites) was randomly selected as training data set and the rest was kept for accuracy assessment. The sample locations are shown in Figure 1.

#### 2.3.4. Land Use Data Processing

We selected paddy fields, dry fields, ditches, woodlands, grasslands, waters, and built-up land from the land use classification data using ArcGIS (ESRI INC., Redlands, CA, USA) software.

#### 2.3.5. Landscape Pattern Index Calculation

The landscape pattern indices of land use data around the snail sites were determined with Patch Analyst version 5, an extended landscape pattern analysis tool of ArcGIS. The landscape pattern indices from landscape analysis are highly concentrated landscape pattern information that can be used to quantitatively evaluate the structural composition and spatial configuration. Landscape metrics can be stratified into six types: area metrics, patch density and size metrics, edge metrics, shape metrics, diversity metrics and core area metrics. A mathematical definition of each metric is detailed in reference [49]. Basing on the ecological significance and avoiding information redundancy, we selected the following landscape metrics: interspersion juxtaposition index (IJI), core area standard deviation (CASD), mean patch fractal dimension (MPFD), patch richness density (PRD), mean proximity index (MPI), total landscape area (TLA) and mean euclidean nearest-neighbor index (MNN).

#### 2.3.6. Significant Factor and Probability Equation Acquisition

The correlative environmental factors of the distribution of *Oncomelania hupensis* were ascertained with a univariate logistic regression model. Independent variables and coefficients were quantified through training data by a forward likelihood ratio (LR) method of the multifactorial binary logistic regression model. Then, an equation for *Oncomelania hupensis* survival probability was obtained. *Oncomelania hupensis* survival probability, namely risk probability of snail habitats, represents the risk extent of a place suitable for snail survival. Receiver operating characteristic (ROC) curve test [50] was used to assess the performance of binary logistic regression model for *Oncomelania hupensis* survival probability. The remaining 25% validation data from both snail-positive (present) sites and snail-negative (absent) sites was used for the purpose.

#### 2.3.7. Potential Habitat Simulation

A predictive risk map of snail habitats was established for the study area based on the probability equation acquired in Section 2.3.6. Potential habitats with risk probability after a flood was extracted from submerged areas and predictive risk map by ArcGIS 10.4.

## 3. Results

### 3.1. Submerged Area after a Flood

**Figure 3:**
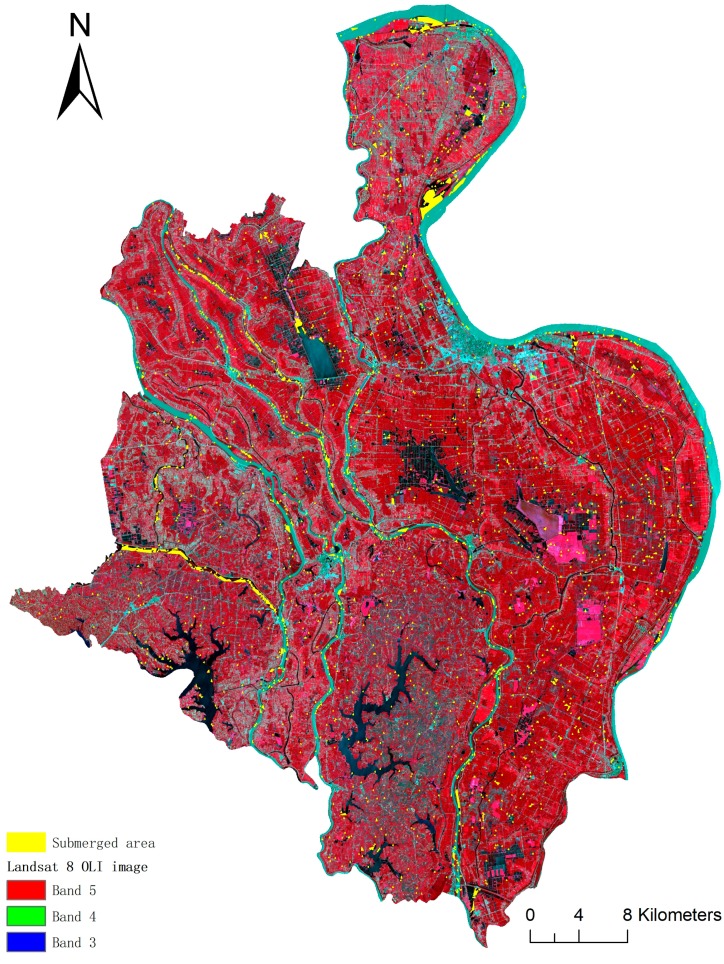
Submerged area.

The submerged areas extracted from Sentinel-1A data are shown in Figure 3. The map shows that most submerged areas were mainly distributed along the river, partly in the fields and ditches. If submerged areas are featured the presence of snails, then these areas are the potential snail habitats after flooding.

### 3.2. Snail Survival and Natural Factors

After univariate logistic regression analysis, the NDVI, wetness index, clay proportion, elevation, land use data and landscape pattern indices were the highly correlated variables inputted into the subsequent multifactorial binary logistic regression model. Then, the forward LR method indicated that the WI (wetness index), NDVI, E (elevation), CP (clay proportion), CASD, WA (woodland area), CLL (construction land length) and DL (ditch length) were the significant risk factors for the presence of snails. The factors exhibiting significant positive correlations were CP, CASD and DL, while the factors exhibiting significant negative correlations were WI, NDVI, E, WA and CLL. The AUC (area under the curve) value of the ROC curve was 0.874 (95% CI: 0.840–0.908), which meant that the logistic regression model could effectively fit the relationship between the environmental factors and snail presence. The final risk probability, *P*, pdiction model of the snail potential habitats after flooding can be represented as follows:

(1) $$p=\frac{1}{1+\mathrm{Exp}\;\left[-(-2.496-5.402\times \mathrm{WI}-1.519\times \mathrm{NDVI}-0.020\times E+0.015\times \mathrm{CP}+0.034\times \mathrm{CASD}-6.216\times \mathrm{WA}-0.229\times \mathrm{CLL}+2.764\times \mathrm{DL}\right)]}$$

### 3.3. Predicted Potential Snail Habitats within the Snail Transmission Reach after Flooding

**Figure 4:**
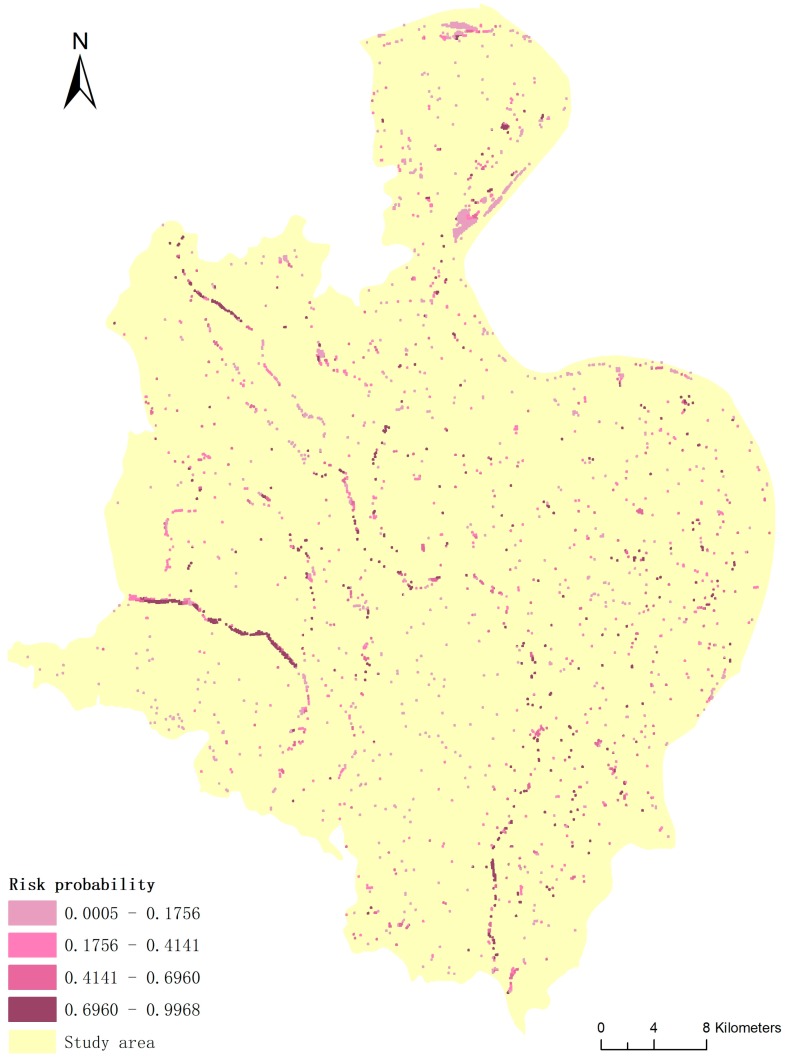
Potential habitats in dispersion ranges of *Oncomelania hupensis* after a flood.

Based on the risk prediction, i.e., Equation (1), we created a 400-m grid with risk probability as cell value to determine the potential snail habitats. Combined the gird and the submerged areas obtained in Section 3.1 (Figure 3), a predictive risk map of snail habitats after flooding was established for the study area. In the map, the deeper the color, the higher the risk of potentially favorable snail habitats. The snails spread to the submerged areas and they grew and bred in the high-risk habitats within these ranges (Figure 4).

## 4. Discussion

### 4.1. The Factors Influencing Snail Habitats after a Flood

The prevalence of schistosomiasis is closely related to natural factors, such as vegetation, temperature, NDVI, humidity, elevation, soil, landscape pattern indices and other factors [51]. However, these factors of snail habitats are complicated and exert combined effects [12], especially in a flood at an unusual time. Typical factors were selected and quantified using binary logistic regression model analysis (Equation (1)). From the results, we determined the significant factors: clay proportion, CASD and ditch length were positively correlated independent variables, while the wetness index, NDVI, elevation, woodland area and construction land area were the negatively correlated independent variables.

The clay proportion was shown to have a positive relationship with snail habitat. The macroscopic distribution pattern of snails depends on the surface features and hydrologic conditions, while the microscopic distribution pattern depends on the vegetation and soil conditions [1]. Soil type is important to *Oncomelania hupensis*. Loamy clay and clay were found to be suitable for snail breeding, whereas sandy loam and sand were not suitable. Therefore, the clay proportion is a good indicator for predicting favorable snail habitat.

CASD also had a positive relationship with snail habitat, indicating that the greater the variability in core area size, the more suitable the environment is for snails. *Oncomelania hupensis* is a type of amphibious freshwater snail that usually grows and breed at the boundary between water and land, such as along ditches, irrigation canals and river banks [1], and the area of water and the area of other land use types usually differ considerably. For example, near ditches, the area of water is usually much smaller than that of land; on river banks, the area of water is usually much larger than that of the river bank. CASD synthesize these differences and can be used to evaluate snail habitats.

Ditch length, which also exhibited a positive relationship with snail habitats, is used as a quantifiable measure of the likelihood of a person coming into contact with snails due to the presence of ditches or canals. The breeding environment of *Oncomelania hupensis* corresponds to areas that alternate between submerged and exposed [52]. Irrigation channel conditions alternate between dry and wet seasonally because the soil moisture and liquid manure levels for crops vary among the different growth stages. These changes regularly coincide with the timing of snail growth and breeding. The snails can spread via water flow in the ditches during periods of irrigation, and can survive in wet soil after irrigation.

The wetness index represents the humidity of the environment and has been shown to be negatively correlated with snail habitat after a flood. Water is one of the necessary conditions for the growth and reproduction of the snails, but the humidity requirements for snail growth are strict and vary among the different stages of growth. Oncomelania is an amphibious snail, and its larva need to live in water. As it grows into the adult stage, it tends to inhabit humid areas, such as grass [53]. Because of its special growing habits, flooding is an unfavorable living condition for adult snails and will inhibit spawning or other reproductive functions, even leading to death. However, flooding promotes the growth of juvenile snails. Additionally, an annual rainfall higher than 1192.2 mm has a negative influence on snails [54]. The density of adult snails on the sides of a channel is greater than that in the center [55]. After a flood, excessive rainfall can increase the environmental humidity. Thus, the wetness index is a negative factor for adult snail habitats.

Appropriate vegetation conditions, which maintain the temperature and humidity, are important for protecting snails from cold in winter and sunshine in summer [56]. The NDVI is a representative index used to detect the vegetation growth status and vegetation coverage. The NDVI appears to have a negative relationship with snail survival, meaning that as the value of the NDVI increases, the snail survival probability decreases. This finding directly opposes those of some previous studies [13,14,17]. One reason is that, spatially, Gongan County is a small-scale study area with many lakes, rivers, ditches, ponds and patty fields. Thus, a high NDVI value usually indicates that the ground is covered with woodland and agricultural land during the vegetative period, whereas a low NDVI value usually indicates that the ground is covered with water bodies or built-up land [48]. Another reason is that, as a rainstorm raises the water level meadows and the sides of ditches become submerged. Therefore, after a flood, lower NDVI values correspond to more suitable environments for snails. In contrast, the NDVI values of ground covered with taller vegetation, such as woods or crops, i.e., areas unfavorable to the survival of the snails, were much higher. Thus, to use the NDVI to study the effects of vegetation on *Oncomelania hupensis*, researchers should also account for the specific ground coverage at the micro-level in the study area.

Elevation also affects the distribution of snails. *Oncomelania hupensis* lives primarily at low elevations, ranging from sea level to 200 m. It inhabits horizontal or nearly horizontal habitats [1]. Most snails usually live within 3 m above the water line along ditches and ponds [56]. From the results, we can conclude that, in a plain area, such as Gongan County, elevation has a negative effect on snails.

Woodland areas and construction land areas appear to have a negative effect on snail habitat. The distribution and reproduction of snails differ among different land uses. Snails are mainly distributed in channels, rivers and the associated paddy fields, ponds, wastelands beside fish ponds and shelter forests [57]. Other areas, such as construction lands, are not suitable for snails due to the lack of water and/or plants. Additionally, the humidity and the distance to waters in these places are not suitable for snail reproduction.

The selection of these particular factors as independent variables for the prediction of snail habitats does not mean that other factors have no influence. The interaction of factors collectively determines the suitability of snail habitats [58]. Numerous environmental factors exert similar effects on snail habitat, and it is necessary to identify representative factors depending on the environment and season.

### 4.2. Potential Habitats of Oncomelania Hupensis in Dispersal Ranges

The area submerged after a flood, as extracted via SAR techniques, is shown in Figure 3. From Figure 3, we can summarize some distribution characteristics of potential snail habitats in Gongan County following a flood. In general, the submerged areas are concentrated in the middle and southeast of Gongan County, which feature low-lying plains and water networks. In the map, nearly half of the visibly submerged areas were along the rivers. In these areas, the common pre-flood boundaries between water and land with snails became submerged during the flood. These submerged areas are considered potential snail habitats because flood-induced inundation of snail-rich areas is usually accompanied by snail dispersal due to the hydrologic regime changes. Being carried by flowing water is the main dispersal method of snails. A large number of snails, especially those exhibiting long-distance migration, spread via flowing water. There are several ways for snails to spread during floods. Snail eggs become exposed when the soft soil covering the surface of the snail eggs gradually dissolves during repeated rain events and ultimately spread via the runoff to surrounding areas or even more distant areas [59]. Very young snails can withstand the flood and live as aquatic snails; thus, they can exploit the surface water currents as a means of locomotion to spread to other places. Adult snails can also take advantage of the water currents by attaching themselves to diverse waterborne objects to spread to new places [52]. Floods can cause significant increases in the snail-positive area in the short term [60], and significant snail migration occurs from upstream to downstream during the flood season in the lower basin of the Yangtze River [15]. Additionally, the spread of snails and schistosomiasis commonly occurs in farmland irrigation systems, such as electrically powered irrigation in a water network region and irrigation systems downstream of a reservoir [61]. The snails spread from snail areas to no-snail areas. When the environment of the no-snail area is suitable, snails will grow and breed quickly, and the snail area will increase rapidly over a short period of time. Therefore, the potential snail habitats in the dispersal range can be determined by delineating the submerged zone.

After determining the surroundings and causes of *Oncomelania hupensis* migration after a flood, we finally simulated and predicted the snail habitats in Gongan County. The risk probability is universally low in the southwestern part of Gongan County, which is characterized by mountains, covered with trees and bushes. Based on a comparison with the land use map (Figure 2), the areas at highest risk are mostly linearly distributed along the rivers, in grasslands and along ditches in paddy fields. The areas with the lowest risk are generally located in construction lands, woodlands, and the interiors of lakes and fields without irrigation canals. The areas with a moderate risk are mainly distributed along ditches in dry fields and occasionally along ditches in patty fields. One reason is that the dispersal range was concentrated in paddy fields with an irrigation system. Implementation of irrigation systems often leads to an expansion of snail habitats, which consequently represent new potential transmission sites for schistosomiasis [2]. The extent of the irrigation system is a strong determinant of the endemic infection levels [62]. Overall, river banks and paddy irrigation ditches represent the most favorable snail habitats after a flood. Adult snails will survive and propagate well along the rivers and ditches. Juvenile snails will then grow in the rivers and be spread to submerged areas via the flowing water. Thus, the rivers and ditches in submerged areas should be a focus of attention.

The potential high-risk snail habitats in the submerged areas should be a focus of attention. The rivers and paddy irrigation ditches are the highest risk habitats for snails in submerged areas. These results will provide scientific guidance for *Oncomelania hupensis* and schistosomiasis control after flooding.

## 5. Conclusions

Floods facilitate the dispersal of snails and can therefore spread schistosomiasis. The potential high-risk habitats within the transmission reach of *Oncomelania hupensis* after floods are worthy of research and identification. Use of Sentinel-1A radar techniques has been shown to be an effective way to identify areas inhabited by snails and submerged due to flooding. We found that the distribution of snails was directly and indirectly related to river systems and paddy irrigation ditches. Based on submerged area map, the potential snail dispersal can be studied. Remote sensing inversion, GIS spatial analysis and statistical analysis are useful in exploring the potential snail habitat and the relevant parameters after a flood. In terms of the suitability of snail habitat, the positively correlated factors are clay proportion, CASD, and ditch length, whereas the negatively correlated factors are wetness index, NDVI, elevation, woodland area, and construction land area. Snail habitats with the highest risk probabilities are mostly linearly distributed along rivers, and along ditches in paddy fields. The rivers and paddy irrigation ditches in the submerged areas are potential habitats with a high probability of snail dispersals after a flood. Accurate simulation of the range of snail dispersal through rivers and ditches is worthy of further research.

## Figures and Tables

**Table 1:** Relevant input data.

Data	Classification
Sentinel-1A data	RS data
Landsat 8 OLI image	RS data
Elevation data	RS data
Snail survey data	Snail data
Village-scale vector map	vector data
Land-use data	vector data
Soil texture data	Raster data

## References

[1] Ross A.G.P., Sleigh A.C., Li Y.S., Davis G.M., Williams G.M., Jiang Z., Feng Z., McManus D.P. (2001). Schistosomiasis in the People’s Republic of China: Prospects and challenges for the 21st century. Clin. Microbiol. Rev..

[2] Steinmann P., Keiser J., Bos R., Tanner M., Utzinger J. (2006). Schistosomiasis and water resources development: Systematic review, meta-analysis, and estimates of people at risk. Lancet Infect. Dis..

[3] Zhou Y.B., Yang M.X., Zhao G.M., Wei J.G., Jiang Q.W. (2007). *Oncomelania hupensis* (gastropoda: Rissooidea), intermediate host of schistosoma japonicum in China: Genetics and molecular phylogeny based on amplified fragment length polymorphisms. Malacologia.

[4] Wu J.Y., Zhou Y.B., Li L.H., Zheng S.B., Liang S., Coatsworth A., Ren G.H., Song X.X., He Z., Cai B. (2014). Identification of optimum scopes of environmental factors for snails using spatial analysis techniques in Dongting Lake Region, China. Parasit. Vectors.

[5] Yang G.J., Utzinger J., Sun L.P., Hong Q.B., Vounatsou P., Tanner M., Zhou X.N. (2007). Effect of temperature on the development of schistosoma japonicum within *Oncomelania hupensis*, and hibernation of *O. hupensis*. Parasitol. Res..

[6] Li J., Zhu X., Zhou G., Cai B. (2002). The compound effect of disastrous floods in Dongting Lake on concurrence of ecological disasters. Acta. Ecol. Sin..

[7] Utzinger J., Zhou X.N., Chen M.G., Bergquist R. (2005). Conquering schistosomiasis in China: The long march. Acta. Trop..

[8] Zhou X.N., Wang L.Y., Chen M.G., Wu X.H., Jiang Q.W., Chen X.Y., Zheng J., Utzinger J. (2005). The public health significance and control of schistosomiasis in China—Then and now. Acta Trop..

[9] Li Y.S., Raso G., Zhao Z.Y., He Y.K., Ellis M.K., McManus D.P. (2007). Large water management projects and schistosomiasis control, Dongting Lake Region, China. Emerg. Infect. Dis..

[10] Wang L.D., Utzinger J., Zhou X.N. (2008). Schistosomiasis control: Experiences and lessons from China. Lancet.

[11] Wu X.H., Zhang S.Q., Xu X.J., Huang Y.X., Steinmann P., Utzinger J., Wang T.P., Xu J., Zheng J., Zhou X.N. (2008). Effect of floods on the transmission of schistosomiasis in the Yangtze River Valley, People’s Republic of China. Parasitol. Int..

[12] Zhang Z., Ong S.H., Peng W.X., Zhou Y.B., Zhuang J.L., Zhao G.M., Jiang Q.W. (2008). A model for the prediction of *Oncomelania hupensis* in the lake and Marshland regions, China. Parasitol. Int..

[13] Yang K., Zhou X.N., Wu X.H., Steinmann P., Wang X.H., Yang G.J., Utzinger J., Li H.J. (2009). Landscape pattern analysis and bayesian modeling for predicting *Oncomelania hupensis* distribution in Eryuan County, People’s Republic of China. Am. J. Trop. Med. Hyg..

[14] Kristensen T.K., Malone J.B., McCarroll J.C. (2001). Use of satellite remote sensing and geographic information systems to model the distribution and abundance of snail intermediate hosts in Africa: A preliminary model for biomphalaria pfeifferi in ethiopia. Acta Trop..

[15] Zhou X.N., Lin D.D., Yang H.M., Chen H.G., Sun L.P., Yang G.J., Hong Q.B., Brown L., Malone J.B. (2002). Use of Landsat TM satellite surveillance data to measure the impact of the 1998 flood on snail intermediate host dispersal in the lower Yangtze River basin. Acta Trop..

[16] Guo J.G., Vounatsou P., Cao C.L., Utzinger J., Zhu H.Q., Anderegg D., Zhu R., He Z.Y., Li D., Hu F. (2005). A geographic information and remote sensing based model for prediction of *Oncomelania hupensis* habitats in the Poyang Lake area, China. Acta Trop..

[17] Zhang Z.Y., Xu D.Z., Zhou X.N., Zhou Y., Liu S.J. (2005). Remote sensing and spatial statistical analysis to predict the distribution of *Oncomelania hupensis* in the marshlands of China. Acta Trop..

[18] Bates P.D., De Roo A.P.J. (2000). A simple raster-based model for flood inundation simulation. J. Hydrol..

[19] Martinez J.M., Le Toan T. (2007). Mapping of flood dynamics and spatial distribution of vegetation in the Amazon floodplain using multitemporal sar data. Remote Sens. Environ..

[20] Barreto T.L.M., Almeida J., Cappabianco F.A.M. (2016). Estimating accurate water levels for rivers and reservoirs by using sar products: A multitemporal analysis. Pattern Recogn. Lett..

[21] Ardhuin F., Collard F., Chapron B., Girard-Ardhuin F., Guitton G., Mouche A., Stopa J.E. (2015). Estimates of ocean wave heights and attenuation in sea ice using the sar wave mode on sentinel-1a. Geophys. Res. Lett..

[22] Grant K., Siegmund R., Wagner M., Hartmann S. (2015). Satellite-based assessment of grassland yields. Int. Arch. Photogramm. Remote Sens. Spat. Inf. Sci..

[23] Wiehle S., Lehner S., Pleskachevsky A. (2015). Waterline detection and monitoring in the German Wadden sea using high resolution satellite-based radar measurements. Int. Arch. Photogramm. Remote Sens. Spat. Inf. Sci..

[24] Yang G.J., Vounatsou P., Zhou X.N., Utzinger J., Tanner M. (2005). A review of geographic information system and remote sensing with applications to the epidemiology and control of schistosomiasis in China. Acta Trop..

[25] Yang K., Li W., Sun L.P., Huang Y.X., Zhang J.F., Wu F., Hang D.R., Steinmann P., Liang Y.S. (2013). Spatio-temporal analysis to identify determinants of *Oncomelania hupensis* infection with schistosoma japonicum in Jiangsu province, China. Parasit. Vectors.

[26] Kitron U. (1998). Landscape ecology and epidemiology of vector-borne diseases: Tools for spatial analysis. J. Med. Entomol..

[27] Turner S.J. (2005). Landscape ecology concepts, methods and applications. Landsc. Ecol..

[28] Brooker S., Hay S.I., Bundy D.A.P. (2002). Tools from ecology: Useful for evaluating infection risk models?. Trends Parasitol..

[29] Van Benthem B.H.B., Vanwambeke S.O., Khantikul N., Burghoorn-Maas C., Panart K., Oskam L., Lambin E.F., Somboon P. (2005). Spatial patterns of and risk factors for seropositivity for dengue infection. Am. J. Trop. Med. Hyg..

[30] Linard C., Lamarque P., Heyman P., Ducoffre G., Luyasu V., Tersago K., Vanwambeke S.O., Lambin E.F. (2007). Determinants of the geographic distribution of puumala virus and lyme borreliosis infections in Belgium. Int. J. Health Geogr..

[31] Ross Z., Jerrett M., Ito K., Tempalski B., Thurston G.D. (2007). A land use regression for predicting fine particulate matter concentrations in the New York city region. Atmos. Environ..

[32] Beelen R., Hoek G., Vienneau D. (2013). Development of NO_2_ and NO_x_ land use regression models for estimating air pollution exposure in 36 study areas in Europe—The escape project. Atmos. Environ..

[33] Long S., Fatoyinbo T.E., Policelli F. (2014). Flood extent mapping for Namibia using change detection and thresholding with sar. Environ. Res. Lett..

[34] Asrar G., Kanemasu E.T., Yoshida M. (1985). Estimates of leaf-area index from spectral reflectance of wheat under different cultural-practices and solar angle. Remote Sens. Environ..

[35] Baret F., Guyot G. (1991). Potentials and limits of vegetation indexes for lai and apar assessment. Remote Sens. Environ..

[36] Richardson A.J., Wiegand C.L., Wanjura D.F., Dusek D., Steiner J.L. (1992). Multisite analyses of spectral-biophysical data for sorghum. Remote Sens. Environ..

[37] Gilabert M.A., Gandia S., Melia J. (1996). Analyses of spectral biophysical relationships for a corn canopy. Remote Sens. Environ..

[38] Leprieur C., Verstraete M.M., Pinty B. (1994). Evaluation of the performance of various vegetation indices to retrieve vegetation cover from avhrr data. Remote Sens. Rev..

[39] Bannari A., Morin D., Bonn F., Huete A.R. (1995). A review of vegetation indices. Remote Sens. Rev..

[40] Elvidge C.D., Chen Z. (1995). Comparison of broad-band and narrow-band red and near-infrared vegetation indices. Remote Sens. Environ..

[41] Danson F.M., Plummer S.E. (1996). Advances in environmental remote sensing. Oceanogr. Lit. Rev..

[42] Amiri R., Weng Q.H., Alimohammadi A., Alavipanah S.K. (2009). Spatial-temporal dynamics of land surface temperature in relation to fractional vegetation cover and land use/cover in the Tabriz urban area, Iran. Remote Sens. Environ..

[43] Chen Y.H., Shi P.J., Li X.B., Chen J., Li J. (2006). A combined approach for estimating vegetation cover in urban/suburban environments from remotely sensed data. Comput. Geosci..

[44] Liu K., Su H.B., Li X.K. (2017). Comparative assessment of two vegetation fractional cover estimating methods and their impacts on modeling urban latent heat flux using landsat imagery. Remote Sens..

[45] Avdan U., Jovanovska G. (2016). Algorithm for automated mapping of land surface temperature using Landsat 8 satellite data. J. Sens..

[46] Kauth R.J., Thomas G.S. The Tasselet Cap: A Graphic Description of the Spectral-Temporal Development of Agricultural Crops as Seen by Landsat. http://docs.lib.purdue.edu/cgi/viewcontent.cgi?article=1160&context=lars_symp.

[47] Crist E.P., Cicone R.C. (1984). A physically-based transformation of thematic mapper data—The tm tasseled cap. IEEE Trans. Geosci. Remote Sens..

[48] Qiu J., Li R.D., Xu X.J., Yu C.H., Xia X., Hong X.C., Chang B.R., Yi F.J., Shi Y.Y. (2014). Identifying determinants of oncomelania hupensis habitats and assessing the effects of environmental control strategies in the plain regions with the waterway network of China at the microscale. Int. J. Environ. Res. Pub. Health.

[49] McGarigal K., Marks B.J. Spatial Analysis Program for Quantifying Landscape Structure. http://www.umass.edu/landeco/pubs/mcgarigal.marks.1995.pdf.

[50] Swets J.A. (1988). Measuring the accuracy of diagnostic systems. Science.

[51] Cross E.R., Bailey R.C. (1984). Prediction of areas endemic for schistosomiasis through use of discriminant analysis of environmental data. Mil. Med..

[52] Zhu H.M., Xiang S., Yang K., Wu X.H., Zhou X.N. (2008). Three gorges dam and its impact on the potential transmission of schistosomiasis in regions along the Yangtze River. EcoHealth.

[53] Wu J.Y., Zhou Y.B., Chen Y., Liang S., Li L.H., Zheng S.B., Zhu S.P., Ren G.H., Song X.X., Jiang Q.W. (2015). Three gorges dam: Impact of water level changes on the density of schistosome-transmitting snail *Oncomelania hupensis* in Dongting Lake area, China. PLoS Negl. Trop. Dis..

[54] Cheng G., Li D., Zhuang D.F., Wang Y. (2016). The influence of natural factors on the spatio-temporal distribution of *Oncomelania hupensis*. Acta Trop.

[55] He L.C., Yuan M.Z., Peng X.W., Dong J., Zhou H.R. (2006). Further observation on snail distribution in ditch water in lake regions. Chin. J. Schist. Control.

[56] Wang J.S., Lu J.Y., Wei G.Y., Yao S.M. (2007). Impact of Environment Changes on Oncomelania Spread. J. Yangtze River Sci. Res. Inst..

[57] Li S.S., Hong H.W., Yu B.X., Liu J., Qin Z.H., Li P. (2007). Research on the molluscacidal effect by concreting ditches for schistosomiasis control in lake regions. J. Public Health Prev. Med..

[58] Remais J., Zhong B., Carlton E.J., Spear R.C. (2009). Model approaches for estimating the influence of time-varying socio-environmental factors on macroparasite transmission in two endemic regions. Epidemics.

[59] Li D.M., Zhan C.H., Hu Z.M. (1997). Studies on Moving of Oncomelania in Water. Adv. Water Sci..

[60] Huang Y.X., Sun L.P., Hong Q.B., Gao Y., Zhang L.H., Gao Y., Chen H., Guo J.H., Liang Y.S., Zhu Y.C. (2004). Longitudinal observation on fluctuation trend of distribution and spread of oncomelania snails after water in marshiland of lower reaches of Yangtze River. Chin. J. Schist. Control.

[61] Wu S.W., Liu X.S., Peng X.P., Xiao J.W., Yao X.M., Zhao Z.Y., Wu Q.Q., Wu M.Q., Pi H., Chen Y. (2001). Study on formative factors attributable to a newly endemic area of schitosomiasis control strategeis within the range of irragation system from Huangshi reseroir. Chin. J. Schist. Control.

[62] Spear R.C., Seto E., Liang S., Birkner M., Hubbard A., Qiu D., Yang C., Zhong B., Xu F., Gu X. (2017). Factors influencing the transmission of schistosoma japonicum in the mountains of Sichuan province of China. Am. J. Trop. Med. Hyg..

